# A Comparative Account of Institutional Approaches to Addressing Campus-Based Sexual Violence in Australia and Aotearoa New Zealand

**DOI:** 10.1177/10778012231183654

**Published:** 2023-07-18

**Authors:** Deanna McCall, Xuan Luu, Chris Krogh, Liam Phelan, Amy Dempsey, Carmen Acosta, Fiona Marshall, Domenic Svejkar, Catharine Pruscino, Melanie A. Beres

**Affiliations:** 1 Student Central, 5982 The University of Newcastle, Callaghan, New South Wales, Australia; 2 School of Medicine and Public Health, 5982 The University of Newcastle, Callaghan, New South Wales, Australia; 3 School of Humanities, Creative Industries and Social Sciences, 5982 The University of Newcastle, Callaghan, New South Wales, Australia; 4 School of Environmental and Life Sciences, 5982 The University of Newcastle, Callaghan, New South Wales, Australia; 5 Campus Community Division, 2541 Monash University, Clayton, Victoria, Australia; 6 Design Innovation Research Centre, 1994 University of Technology Sydney, Sydney, New South Wales, Australia; 7 Office of the Provost, 1994 University of Technology Sydney, Sydney, New South Wales, Australia; 8 Te Whare Tāwharau, 2495 University of Otago, Dunedin, Otago, New Zealand

**Keywords:** sexual violence, prevention, higher education, whole of university, qualitative‌

## Abstract

Sexual violence is prevalent on university campuses globally. In this article, we report a qualitative insider research study examining practices for addressing sexual violence at four universities across Australia and Aotearoa New Zealand. We collected, analysed, and synthesised descriptive information about the practices at each institution. We found unique institutional approaches that nonetheless share some commonalities, yieldingseveral themes that are central to practice. In reflecting on our findings, we conclude with an outline of critical considerations and a call to action for future efforts to address campus-based sexual violence, particularly as this field remains underdeveloped across Australia and Aotearoa New Zealand.

## Introduction

Sexual violence is a serious public health, human rights, and social justice concern across universities internationally, including in Australia and Aotearoa New Zealand. Research exploring the prevalence and impact of campus-based sexual violence has grown internationally since the 1950s (Jessup-Anger et al., 2018). Much of this work has emerged from North American higher education institutions, framed by two key paradigm shifts: (a) rising student-led advocacy and activism against campus-based sexual violence (Krause et al., 2017) and (b) increasing critical attention from media and society on how universities respond to the issue across their campuses (Amar et al., 2014; Richards, 2019). These shifts have sharpened the focus on preventing and responding to campus-based sexual violence.

By comparison, across Australia and Aotearoa New Zealand, efforts have been ad hoc. Universities have addressed campus-based sexual violence in institution-specific, location-specific, and case-specific isolation, with reluctance to formally recognize the issue and establish sustained, integrated responses (Durbach & Grey, 2018). This has rendered absent a shared framing of the issue and a clear sense of purpose at the regional level.

### Background

In 2016, the Australian Human Rights Commission (AHRC) conducted a landmark national survey on campus-based sexual violence. The survey sought to better understand students’ self-reported experiences of two distinct types of sexual violence: sexual assault and sexual harassment (SASH). The sample comprised approximately 30,000 students across domestic, international, undergraduate, postgraduate, coursework, and higher-degree research cohorts. The resulting report, *Change the Course* (AHRC, 2017), detailed findings from the national survey and presented nine recommendations for action.

*Change the Course* highlighted the prevalence of sexual violence across Australian universities. Approximately half (51%) of survey respondents reported at least one experience of sexual harassment in 2016, and approximately 7% of respondents reported at least one experience of sexual assault in 2015 or 2016 (AHRC, 2017). Further, an overwhelming majority of respondents who had experienced SASH did not make a formal report or file a complaint (94%), nor did they seek any assistance or support from their university (92%) (AHRC, 2017). At the time of writing this article, national sector data are not available for Aotearoa New Zealand. Through their study of one university campus, Beres, Stojanov et al. (2020) found rates of campus-based SASH consistent with international research: 28% of respondents reported experiencing some form of sexual assault during their time at university, and 60% of respondents reported experiencing at least one form of sexual harassment from another student.

Both *Change the Course* and the study by Beres, Stojanov et al. (2020) highlighted the extent to which students with diverse identities are disproportionately affected by campus-based sexual violence. Women and non-binary students, students from ethnic minority groups, students identifying as LGBTIQA+, and students with a disability reported experiencing higher rates of SASH at university when compared with other students—for example, men who are heterosexual, cisgendered, and from dominant cultural groups (AHRC, 2017; Beres, Stojanov et al., 2020). These impacts reflect rates of sexual violence among disproportionately affected groups in broader society (for example, see Australian Institute of Health and Welfare, 2019).

### Recommendations for Action

*Change the Course* outlined nine recommendations, including one specific to residential colleges, that aim to drive change across several action areas. These action areas are purposeful leadership, addressing attitudes and behaviors, ensuring reporting and response pathways, adopting strategies that have been shown to work and continuing to build the evidence base, and addressing factors in residential college settings. The action areas implicate two key determinants of sexual violence that operate in university settings: (a) culturally embedded views and expectations surrounding gender, sex, entitlement, and power and (b) institutional and societal structures that reinforce ongoing oppression and marginalization of specific groups.

### A Whole-of-University Approach

The action areas also highlight the importance of a whole-of-university approach. In addressing complex health and social issues—including sexual violence—this type of approach involves the entire university system, bringing together students, staff, senior leadership, and surrounding communities (Dooris et al., 2020). In so doing, it draws on two viewpoints. First, multiple concurrent interventions are better than singular standalone interventions. Second, the university setting is considered from a social-ecological systems perspective, presenting opportunities for campus community involvement to achieve meaningful change. These two viewpoints have informed other published scholarship on campus-based sexual violence (for example, see McMahon et al., 2019; Moylan & Javorka, 2020).

Multiple interventions are essential for addressing campus-based sexual violence. Examples of these are transparent policy measures, trauma-informed supports in place for those impacted, clear pathways for reporting and for formal investigations, and evidence-informed strategies for primary prevention (Beres, Treharne et al., 2019). To create conditions that enable these interventions, universities must act throughout their social-ecological systems—including changing institutional norms and policy, engaging campus community members, involving community voices throughout intervention lifecycles, and building on both strengths and risk factors in their contexts (Banyard et al., 2019; DeGue et al., 2016; Elton et al., 2019; Perkins & Warner, 2017). As such, in whole-of-university approaches, the whole is greater than simply the sum of its parts.

### Research, Practice, and Policy Context

The release of *Change the Course* prompted new attention across research, practice, and policy. Region-wide conferences on addressing campus-based sexual violence are one form of this growing attention (for example, Australasian Universities Safer Communities Symposia, Australia and New Zealand Student Services Association conferences, Respect. Prevent. Respond. conferences, and University of Otago-hosted symposia). Growth in scholarly literature (for example, see Byfield & East, 2018; Elton et al., 2019; Henry, 2019; Irvine-Collins et al., 2022; Ison et al., 2022; Showden, 2018; McCall et al., 2020; Wills & Duncan, 2018) and practice guides (for example, see Durbach & Keith, 2017; Universities Australia, 2018) is another. Higher education regulatory agencies have begun to integrate addressing campus-based sexual violence into standards that assure students’ safety and wellbeing (for example, see Tertiary Education Quality and Standards Agency, 2020). Peak bodies have also instituted sector-wide campaigns, such as Respect. Now. Always. in Australia (Universities Australia, 2017). These developments create fertile ground for a cross-sectional view of practices, grounded within the wider landscape of existing frameworks and evidence.

### Aims and Purpose

We are a multidisciplinary collective of professional and academic colleagues affiliated with multiple universities across Australia and Aotearoa New Zealand—in particular, four institutions that pursue whole-of-university approaches to addressing sexual violence. Herein, we describe and discuss practices enacted at those four institutions. We seek to answer the following question: “What are some of the similarities and differences in addressing campus-based sexual violence across these institutions, and what have we learned from our experiences in carrying out this work?”

Our intentions are threefold: (a) to detail our exploratory findings, (b) to discuss commonality and diversity in practices, and (c) to highlight areas of strength and vulnerability in this work. To do this, we begin with an overview of the approach at each institution. We then synthesize practices across the four institutions by building key themes, identifying commonalities and differences, and reflecting on our findings. We conclude with critical considerations and a call to action forfuture research, practice, and policymaking.

## Research Approach and Findings

We adopted a qualitative insider research approach (Berkovic et al., 2020; Greene, 2014; Kinitz, 2022). We created a structured template to purposively collect descriptive information from (a) Monash University, (b) the University of Newcastle, (c) the University of Otago, and (d) the University of Technology Sydney. For each institution, we mapped practices against four domains inherent to whole-of-university approaches: (a) policy development, (b) support, (c) education and prevention, and (d) reporting and investigations. Table 1 presents an overview of the practices reported. Given the inevitability of nuances specific to our region, Appendix A provides a glossary with explanatory detail.

**Table 1.** Overview of Institutional Practices.

Domain of action	Students	Staff	Senior leadership	Broader community
**Monash University**				
*Policy development*	Overseen by Respect. Now. Always. Advisory Committee, comprising student leaders, staff (academic and professional), and senior leadership			Consultation with local community-based expert organizations (for example, the South Eastern Centre Against Sexual Assault)
*Support*	Establishment of a dedicated Safer Community Unit, with specialist trained staff, to respond to disclosures and reports		Governance of resourcing, monitoring, and review	South Eastern Centre Against Sexual Assault-employed counselors situated within University Counseling Services for students and staff
	Responding to disclosures training available to student leaders and staff to support victim/survivors			
*Education and prevention*	Education and training focused on on respectful relationships, active bystander intervention, and the drivers of gender-based violence	Online module for staff with research supervision responsibilities titled “Respectful Supervision of Students” On-request training for specific groups (for example, Flip the Script—Enhanced Assess, Acknowledge, Act [EAAA])	Governance of resourcing, monitoring, and review	External expert speakers engaged ad hoc for events and activities
	Mandated training for all newly enrolling students and all new on-campus residents			
	Communications and promotion to promote key services			

* Reporting and investigations*	*Sexual Misconduct Response Procedure*	*Behaviors in the Workplace Procedure*	Receive annual detailed data on disclosures and reports received by the University	Availablity of systems that enable public reporting of SASH to the University
	The Safer Community Unit coordinate investigations and the Office of Student Conduct coordinate decision-making			
		Ethical Conduct staff within the University's Human Resources department		
**The University of Newcastle**				
*Policy development*	Initially established a Sexual Misconduct Working Group which has been since beenreplaced by the Safe and Respectful Communities Working Group, bringing togetherkey internal informants (student leaders, staff, and individuals with lived experience of SASH)		Resourcing and delivering open forums across campus communities	Consultation with community-based organizations
*Support*	24-hour crisis support line available since 2017, including online, face-to face, and telephone options for any university community members wishing to disclose or submit a report	Campus Care, Student living Support team, Security	Governance of resourcing, monitoring, and review	Working relationships with external services such as the state-level New South Wales (NSW) Sexual Violence Helpline and the national-level Full Stop Australia organization
		Employees Assistance Program (EAP) for staff and their families to access psychological services		
	Trauma-informed counseling services for students			
	Student support staff with specialist training to support students who have experienced SASH, and aRespectful Communities Coordinator role focusing on secondary prevention through referral to university support services			
		Delivery of Full Stop Australia's evidence-informed *Sex, Safety & Respect *program with staff		
	Mandatory online consent education module for all students Consent 'labs' coordinated by the Student Living department during Orientation Week	Modules and capacity-building sessions focusing on responding to disclosures and on technology-facilitated abuse	Orientation programs focused on SASH and affirmative consent organised by the Student Living department	Engagement of community-based organisations, such as Full Stop Australia, to train staff in the delivery of prevention education with students Partnership with Epigeum and leading experts across the United Kingdom and Australia, resulting in development of an online module to support staff and student leaders in responding to disclosures of sexual violence
*Education and prevention*	Delivery of Full Stop Australia's *Sex, Safety & Respect* program with students			
	University-wide “No Room 4” student engagement initiative, underpinned by principles of changing social norms through social marketing			
	Respectful Communities Coordinator role focusing on primary prevention through education and capactiy-building			
*Reporting and investigations*	Anonymous and identified reporting pathways	Campus Care staff oversee investigations and ensure consistent policy implementation	Completion of mandatory online consent education module	
			Participation in external and cross-institutional reviews	
**The University of Otago**				
*Policy development*	Student-driven demand for policy change	Two working groups who developed policy and response	Vice-Chancellor and Senior Leadership Team put sexual violence on agenda due to international attention to the issue	Consulted with community-based groups and organizations
	Student involvement in committees that led to policy change			
*Support*	Dedicated center for support	Dedicated center for support		External referrals for longer-term support
	Student volunteers for peer support	EAP services		
*Education and prevention*	Social marketing campaign	Training on responding to disclosures and on relevant university policies, embedded into commencement processes for new staff		
	Four workshops for student cohorts: (1) incoming students in residence, (2) student leaders, (3) bystander, and (4) EAAA			
*Reporting and Investigations*	Reporting pathways through the university proctor or through the university's support services	Proctor, who oversees campus security and other disciplinary measures, undertakes investigations		
		Team of staff oversee investigations and ensure consistent policy implementation		
**The University of Technology Sydney **				
*Policy development*	Respect. Now. Always. SteeringGroup comprisingstudent leaders, staff, and senior leadership			Consultation with community-based organizations and subject matter experts, such as Full Stop Australia
	Consultation with student leaders, practitioners, and subject matter experts to ensure that evidence and best-practice inform policy and procedure		Commissioned three university-centered, campus community-based research projects and reports	
			Student Voice research project initiated to inform strategic direction	
	Reviewed, and updated where appropriate, policies and procedures—including the *Equity, Inclusion and Respect Policy*, the Student Rules, the *Code of Conduct*, and the *Graduate Research Supervision Policy*			
			Establishment of a university-wide strategic framework to drive longer-term sustainable cultural and systemic change	
	Developed and implemented a new *Sexual Harm Prevention and Response Policy*, supported by a new cross-sectional governance structure			
			Exploration of the barriers and enablers that support positive culture change within the university	
*Support*	24-hour crisis support line available since 2017, including online, face-to-face, and telephone options for any university community members wishing to disclose or submit a report			Close working relationship with the local NSW Police Area Command where needed
	Trauma-informed psychologists on staff to provide support for students			
	EAP for staff and their families			
	Annual completion of Full Stop Australia's Responding with Compassion training by staff in the Security Services department			
*Education and prevention*	Tailored communication strategy promoting available support and resources within and outside the University			External community-based speakers and educators engaged for training, events, and activities
	Student-focused awareness campaign throughout the Orientation period	Good Night Out training for all university bar staff	Governance of resourcing, monitoring, and review	
	Mandatory online consent education module	Mandatory online consent education module	Mandated online consent education module	
			Senior leaders advocate for, and present on, the work of the Respect. Now. Always. team, on expectations of staff, and on available resources and support for the whole campus community	
	Delivery of Full Stop Australia's *Sex, Safety & Respect* training with all student leaders in clubs, societies, and university accommodation, incuding provision to the wider student population by upon request			
		On- request training for staff groups, including foci such as (active bystander intervention training, vicarious trauma, and Mental Health First Aid)		
		Student-facing staff from across the university trained to deliver Full Stop Australia's *Sex, Safety & Respect* with students		
	Development of a planning and response toolkit for student club executive members for all overnight events			
	Respect. Now. Always. Crew—a volunteer group of students and staff who operate as local-level champions of sexual violence prevention and response, helping to lead activations, training, events, and research			
*Reporting and investigations*	Online, face-to-face, and telephone options for any university community wishing to disclose or submit a report			
	Verification that students have completed the mandatory online consent education module, as this is noted on students’ Australian Higher Education Graduation Statements (AHEGS)	Staff completion of the mandatory online consent education module is reported on a monthly basis to members of senior leadership	Receive, and make decisions about, monthly data on onlinie mandatory consent education module completion rates among all staff	
			Receive, and make decisions about, annual data on SASH-related disclosures and reports received by the university	
	Student Complaints department			
			Reports on the progress and achievements of the University's Respect. Now. Always. Program teamare provided to the University Council and Academic Board	
	Misconduct department			

To contextualize the information presented in Table 1, we provide below a narrative account of the efforts at each institution. For each account, we begin with an outline of institutional characteristics, and we then document the institution's efforts up to the time of writing this article. We subsequently adopt a reflexive approach—informed by principles of thematic analysis (Braun & Clarke, 2006, 2019; Braun et al., 2022)—to identify and discuss key themes that are central for practice.

### Monash University

Monash University is a large Australian university, comprising four campuses in metropolitan locations with several smaller teaching sites locally and abroad. Approximately one-third of the student population are international students, and most students are undergraduate. Although mechanisms and pathways to respond to campus-based sexual violence have existed at Monash since 2008, *Change the Course* drove a much-needed additional focus on prevention. This led to the establishment of a university-wide Respect. Now. Always. Advisory Committee in 2018 with two remits: (a) to address all recommendations from *Change the Course* and (b) to provide oversight of prevention and response initiatives across the institution. The Committee pursued a comprehensive approach by formalizing a team of specialist staff, called Respectful Communities, to lead the institution's whole-of-campus prevention efforts.

Throughout 2018, Monash University focused on educational and awareness-raising initiatives with newly enrolling students, including an elaborate face-to-face orientation campaign preceded by an online module on respect. Since 2019, students who do not complete the module have had an encumbrance placed on their record, preventing them from accessing course results and other institutional systems. This represents a systematic, structural intervention to enhance education for prevention.

Moreover, in line with *Change the Course* recommendations, Monash University implemented initiatives aimed at addressing drivers of SASH specific to students living on campus. This included social norms marketing and training programming, with a 1-hour, face-to-face, peer-led session on consent and respectful relationships. The institution also implemented externally facilitated training for student leaders and staff, led by community-based expert victim/survivor advocates and counselors, with a focus on responding to disclosures of sexual violence. Following the training of several hundreds of student leaders and staff across major campuses, initial evaluation demonstrated a need to expand training to ensure staff and student leaders across the institution at large—both local and overseas—could participate. The training was then translated in 2019 to an online module format, guided by expert advice again from community-based expert victim/survivor advocates and counselors. This has expanded uptake of the content significantly among staff, particularly at international campuses and sites.

After addressing recommendations from *Change the Course*, Monash University focused prevention efforts on refining existing education interventions and introducing anew. In 2019, Monash University launched an updated student leadership training program focused on bystander intervention, respect, intimate partner violence, and gender inequality, implemented with almost 1,000 students. This program has become mandatory for student leaders across student organizations—including clubs and societies—and residential settings.

The Respectful Communities team adopts a peer-led model. Currently enrolled students are recruited, engaged, and trained as casual staff to deliver all student-focused training, as well as to engage with campus community members during awareness-raising events. This model includes strong, ongoing working relationships with student organizations. The team provides advice and support to student leaders who seek to deliver events relating to sex, relationships, affirmative consent, and sexual health. The team also partners with students to design projects that align with broader diversity and inclusion priorities across the institution.

In 2019, Monash University piloted the evidence-informed EAAA Sexual Assault Resistance program, building on the program's evaluated effectiveness in reducing campus-based sexual violence (for example, see Senn et al., 2015). The Respectful Communities team also developed an evidence-informed program to provide a supportive environment in which men students could challenge stereotyped constructions of masculinity and femininity as determinants of violence against women.

As part of their whole-of-campus approach, Monash University also embedded prevention within policymaking through a university-wide *Sexual Misconduct Response Procedure*. While this policy intervention was designed to articulate how the institution manages disclosures and reports of sexual misconduct by students, it also included a clear statement of the institution's commitment to a range of prevention initiatives.

In 2020 and beyond, Monash University expanded more tailored programming led by the Respectful Communities team. This tailoring approach enabled outreach across the whole institution, including community members at international campuses. It also informed the provision of more online resources on addressing campus-based sexual violence for students and staff, with a particular focus on sensitivity to local cultural contexts.

In contributing to a sector-wide understanding of “what works,” Monash University continues to refine its evaluation. To date, evaluation has largely comprised empirical designs and methods, such as pre-posttest surveys and focus group discussions. The Respectful Communities team continues to develop relationships with academic colleagues and external experts to further strengthen their mixed-methods evaluation. For instance, in 2020, the team launched its program engaging men students in gender-based violence prevention and in so doing partnered with academic staff to evaluate its impact (see Elliott, et al., 2022). While considerable progress has been made, Monash University recognizes a long road ahead in continuing to address campus-based sexual violence—including learning what leads to more effective prevention and response.

### The University of Newcastle

The University of Newcastle is an Australian university with two primary campuses and multiple satellite sites, including one medium-sized regional campus. Its student population is primarily undergraduate (79%), with relatively fewer international students (17%) compared to other Australian universities. The institution significantly revised its policies and practices in response to *Change the Course*.

In 2017, the University of Newcastle established a cross-sectional working group focused on addressing sexual misconduct. This group comprised professional staff, academic staff, and student representatives. As a governance body, the group engaged with external specialist agencies to develop two structural changes: (1) a new university-wide policy and associated procedure specific to responding to SASH, and (2) an organizationally centralized pathway for reporting incidents and making disclosures of sexual violence affecting students. Guided by principles and standards documented in the former, the latter was designed with two functions: (1) to facilitate both identified and anonymous reporting, and (2) to enable support for students in supporting any peers impacted. Reports received are assessed initially by licensed mental health clinicians within the institution's student services department, with consideration of risk, safety management, and referrals to additional support services as needed. The assessment approach emphasizes the safety and wellbeing of the person affected.

In 2017, the University of Newcastle introduced an online consent education module for students living on campus. In 2018, following advocacy and support from the student population, the institution mandated this module as a term and condition of enrolment for newly commencing students. Since 2017, all staff have had access to online resources focused on image-based abuse and responding to disclosures of sexual violence. Clinicians in student support roles complete specialist training on trauma-informed care, framed by provision of an after-hours telephone line to enable crisis support for students. In 2019, this suite of interventions formed part of a broader whole-of-campus campaign on effective, sensitive, and coordinated approaches to addressing sexual violence and other forms of interpersonal harm.

In 2018, the University of Newcastle partnered with an external specialist organization to train 20 staff—including counselors, wellbeing services staff, student accommodation staff, and academics—in delivery of the organization's flagship prevention programming. The programming addressed ethical interpersonal negotiation and communication, consent and the law, and ethical bystander intervention. Since 2019, these trained staff have facilitated workshops with all newly incoming students in on-campus residences. Workshops are also incorporated into specific coursework degrees, including social work and teacher education, representing a curricular infusion intervention. Evaluation of these educational interventions adopts a pre-posttest design with a planned follow-up at 12 months post-intervention.

### The University of Otago

The University of Otago is located in a small city in Aotearoa New Zealand. Most students study at the main campus, with the majority attending for undergraduate study and an approximate proportion of 15% international students. In 2016, the institution was driven to change by two factors: (a) increased senior leadership awareness of international attention on the issue of campus-based sexual violence, and (b) increased student group campaigning for university responses to their concerns about sexual violence. Following development and consultation since 2016, the institution released its university-wide policy on sexual misconduct in 2019 with a revision in 2022. Investigations into sexual misconduct are handled by the institution's proctor, involving a team of approximately 10 professional and academic staff.

The University of Otago adopts a whole-of-university approach through collaborative engagement with students, professional and academic staff, and community-based organizations. The approach builds on principles that include an evidence base for best practice, integrated and holistic practice to ensure consistent responses to sexual misconduct across the institution (including residential colleges), and student and staff involvement. The institution also includes a center dedicated to the leadership of prevention efforts and to the provision of support for those impacted.

Three peer-led prevention programs are delivered with students on campus: (a) the EAAA program, (b) Bringing in the Bystander (see Soteria Solutions, 2020); and (c) CommUNIty102, a consent- and alcohol-focused program. CommUNIty102 is delivered to all incoming students through the residential colleges, amounting to approximately 3,000 students per year. Students residing locally are also invited to attend. The center supplements programs with additional linked initiatives that include social marketing campaigns, staff training, support for victim/survivors, and disciplinary procedures.

All staff at the University of Otago are invited to seminars on how to respond to disclosures of sexual violence, including a focus on their roles and responsibilities as framed by the institution's policy on sexual misconduct. Training is integrated into induction for all new campus security staff and specific staff in residential colleges. Support services also provide capacity-building for staff in teaching about sexual violence and caring for students.

### The University of Technology Sydney

The University of Technology Sydney is a mid-sized Australian university located in the central business district of a capital city. Approximately one-third of their student population are international students, and they focus strongly on undergraduate enrolments (72%). The institution includes one primary campus and several specialist facilities off-site. The institution's Provost, on behalf of the Vice-Chancellor, has accountability for a university-wide Respect. Now. Always. program focused on addressing sexual and gender-based violence. A dedicated program manager leads this work, supported by a high-level, cross-sectional senior working group comprising representatives from student bodies and the institution's main operational units. The program works toward stronger coordination and improvement of governance, community-focused prevention, and trauma-informed support, embodying implementation of an institution-wide cultural change framework.

The University of Technology Sydney implements a community-based approach. In 2018, the institution delivered its Student Voice research report, synthesizing contributions from over 3,000 students and staff on the issue of campus-based sexual violence. As an output of community-based inquiry, the report documented a granular understanding of students’ self-reported experiences of campus-based sexual violence. It outlined 21 key insights and discussed implications for the institution in its future efforts to prevent SASH.

As a result of its research and community consultation, the University of Technology Sydney has implemented a 24-hour telephone support line for those affected by sexual assault, integrated trauma-informed staff training, a mandatory online consent education module for all students and staff with over 65,000 completions as of late 2020, and establishment of a Student Consultative Group to promote student-centered advocacy and decision-making on institutional action. University-wide messaging has emphasized support services within and beyond the campus community.

The University of Technology Sydney adopts a long-term approach to addressing sexual violence, focused on sustainable cultural and systemic change, and enacted at whole-of-university scale. This involves understanding the attitudes giving rise to gender inequality and addressing behaviors that contribute to sexual violence. Their approach has coevolved activities with students and staff over several years, including community-led activations. These activations aim to facilitate and maintain safe, fun, and accessible conversations about consent, respect, inappropriate behaviors, and sexual violence. Led by a growing network of student and staff volunteers, they help to normalize discussions about sexual violence, unequal power structures, and attitudes that perpetuate and reinforce SASH. In 2020, the University of Technology Sydney's Respect. Now. Always. program was recognized by Good Design Australia with a Best in Class (Social Impact) award for whole-of-community sexual violence prevention (see Good Design Australia, 2020).

To further drive long-term, sustainable cultural change at a whole-of-campus scale, the University of Technology Sydney commissioned a second research project to develop a whole-of-community policy intervention—its Respect. Now. Always. Strategic Framework released in 2019. Working with students and staff as subject matter experts across the institution, the research aimed to better understand how SASH presents within the institutional community and to codesign a way forward. The framework is currently in implementation, working with groups of students and staff across the institution to determine how they can integrate sexual violence prevention and response into their teaching, research, and practice. This policy intervention is strengthened by recent institutionalization of a new university-wide Sexual Harm Prevention and Response Policy, which is further supported by a new cross-sectional governance structure.

### Key Themes for Practice

Our research demonstrates that whole-of-university approaches to addressing campus-based sexual violence comprise a breadth of interventions. In gathering, synthesizing, and reflecting on practice-based information across the four institutions, we constructed several themes that can be considered central to practice: a multifaceted approach, leadership, inclusivity and internal collaboration, external partnerships and collaboration, supportive responses and provision of support services, and evaluation. In this section, we discuss each theme in greater detail, with reference to relevant examples of practice.

#### A Multifaceted Approach

To address campus-based sexual violence, a multifaceted approach increases methods of engagement and participation throughout campus communities. It also guides universities in adopting multiple concurrent interventions for prevention and response. We found that all four institutions implemented face-to-face preventing programming with students. All four institutions also implemented training and information on responding to disclosures of sexual violence with staff. These two interventions were typically combined with additional measures such as online awareness-raising, social marketing campaigns, campus-wide visibility through stalls and merchandise at university events, public-facing events, volunteer networks, and staff mentoring. Multifaceted approaches contribute to consistent and persistent messaging across campus environments, which in turn can support changes to institutional norms.

#### Leadership

Institution-wide cultural change is a long-term work that necessitates effective leadership. We found this to be a prominent consideration across all four institutions. Attention to leadership means extending focus beyond simply the content and practice of initiatives; consideration is also needed of the wider contexts in which these initiatives operate. Leadership of campus-based sexual violence prevention and response efforts is clear at all four institutions—such as at the level of Vice-Chancellor (Monash University and the University of Otago), Deputy Vice-Chancellor and Provost (the University of Technology of Sydney), and Pro Vice-Chancellor (the University of Newcastle). We argue that clear, committed senior leadership is essential for contributing to universities’ efforts in addressing campus-based sexual violence.

Leadership is also crucial for formalizing institution-wide commitment. Through this, universities can establish consultative structures and practices to support change. Examples include institution-wide governance groups (such as the Respect. Now. Always. Advisory Committee at Monash University and the Senior Working Group at the University of Technology) and victim/survivor-led approaches to changing measures in policy, support, and prevention (such as at the University of Otago).

We also note the importance of leadership dispersed throughout institutions. Distributed leadership (for example, see Carbone et al., 2017; Spillane et al., 2004) posits that leadership can unfold across levels of seniority and across contexts—above and beyond formal leadership roles. This is clear across the four institutions, where many student and staff collectives have led efforts. Capacity-building for effective leadership in addressing campus-based sexual violence is therefore critical.

#### Inclusivity and Internal Collaboration

Universities bring together different voices, perspectives, and values, calling for spaces in which they can be seen, felt, and heard. In addressing campus-based sexual violence, inclusivity can take on many forms. For instance, it can emerge through bringing international students together in conversation about consent and sexual violence, with sensitivity to ethnoculturally ingrained understandings. It can manifest in awareness-raising about sexual violence with men students, aiming to help them feel part of cultural change—rather than ostracized or demonized. It can also draw staff together as they talk about, and make sense of, how institutional power can overtly or covertly shape sexual violence. At the heart of these practices is collaboration, cutting across the hierarchical structures and boundaries so commonly etched in university settings.

Across all four institutions, we found approaches that involve strong partnership between students and staff. Student-staff partnership aims to reposition students and staff as active collaborators in higher education practice (Mercer-Mapstone et al., 2017). In so doing, it challenges conventional hierarchical norms in institutions (Mercer-Mapstone & Bovill, 2020).

Student-staff partnership takes different forms across the four universities. For example, at Monash University, prevention programming involves students in peer-led delivery that focuses on education and awareness-raising with specific cohorts (for example, EAAA with women students, programming with men and gender-diverse students, and student-led consent and sexual health webinars with international students). The University of Newcastle has fostered partnership through a student-led, campus-wide campaign based on research with young people and aiming to provide a clear platform for student voices against unacceptable behaviors; this campaign was codeveloped with contributions from staff working in student accommodation settings. The University of Otago's tailored programming ensures staff collaboration with Māori and Pacific students, with particular focus on a dedicated Pacific Student Navigator role that partners with students in delivering robust prevention programming and support. Moreover, the University of Technology has adopted a participatory codesign approach with students and staff across the institution—including workshops focused on goals and challenges in the field of campus-based sexual violence and codevelopment of initiatives and strategies for community-wide implementation.

#### External Partnerships and Collaboration

We found that all four institutions engaged with broader, external, community-based efforts to address sexual violence. Strong working relationships with external experts and services were found to be key. For instance, all four universities reported developing ongoing relationships with local stakeholders and services related to sexual violence response—including police when needed—to inform institutional responses on campus.

There continues to be great potential in external partnerships to further how universities address campus-based sexual violence. Public transport providers are one such example. *Change the Course* noted a high incidence of sexual violence—particularly sexual harassment—affecting students on public transportation to and from university. Local councils and hospitality businesses are further examples, given their oversight over locations proximal to campus environments. Through these additional partnerships, universities can strengthen their capacity and impact—from cross-sectoral initiatives to codesigned research and evaluation.

Partnerships across multiple higher education institutions are also instrumental in addressing campus-based sexual violence. They create contexts for conversation and collaboration across institutions, pursuing goals such as sharing ideas, learning from common experiences, and collectively developing resources. Conferences and symposia are one such example. Our article, bringing together authors from multiple institutions with a shared vision for safer campus communities, is another. It is vital that these opportunities continue to grow; this would ensure that institutional silos can be dismantled through collective effort.

#### Supportive Responses and Provision of Support Services

Recent research highlights how sexual violence can negatively impact academic outcomes among student victim/survivors (Potter et al., 2018). As such, universities have recognized their critical role in supporting affected students. Across the four institutions, we found structural interventions—including changes to policies, procedures, and reporting pathways—designed to improve supportiveness and person-centeredness. The streamlining of reporting pathways can minimize the need for victims/survivors to retell their story multiple times (Lievore, 2005). It can also provide a means for disclosures to be victim/survivor-led rather than necessarily compelled (Holland et al., 2018). This work reflects trauma-informed practices, involving sensitivity to the rights of a victim/survivor in controlling how they wish to proceed with disclosures of their lived experiences (CASA House, 1990, 1993).

We found that all four institutions have supplemented structural change with capacity-building to improve interpersonal support provision. This work has included implementation of resources and training in areas including establishment of informal support networks and pathways, skill-building in responding to disclosures of sexual violence, and instilment of support services specific to sexual assault (for example, a telephone line for crisis support or a dedicated center for prevention and response). We also found further recognition of the need for additional care to support frontline responders working directly with those impacted.

#### Evaluation

Evaluation is key throughout the intervention lifecycle—beginning from the earliest stages of development and implementation—for different purposes and using different methods (Grinnell et al., 2012). It can clarify benefits and challenges. In so doing, it can guide decision-making on how to extend, adjust, or perhaps even abandon delivery over time.

We found evaluation efforts across all four institutions. Monash University, the University of Otago, and the University of Technology integrated evaluation into their planning and implementation of interventions. This included formative review of evidence on prevention practices, community engagement to assess perceptions of existing support and reporting pathways, and adoption of a systems-thinking view to interrogate how components of whole-of-campus approaches can work together. The University of Newcastle also conducted impact evaluation of their prevention programming with a planned longer-term follow-up at 12 months.

Most importantly, we acknowledge that while there is value in cross-institutional evaluation, the considerable diversity and lifespan of interventions are barriers to comparing impacts and outcomes across multiple universities. This complexity is compounded by a continuing lack of localized scholarship on addressing campus-based sexual violence specific to our region. Nonetheless, we argue that dissemination of shared learnings, experiences, and practices contributes to a better understanding of what works—and what works well.

## Discussion

Through our research, we have explored whole-of-campus approaches to addressing campus-based sexual violence throughout four institutions of higher education across Australia and Aotearoa New Zealand. We recognize that much of this work reflects learnings from a rich history of research, practice, and policymaking in North American colleges and universities—as we acknowledged at the outset. We also note that institutions have taken steps to elevate the voices of students from marginalized communities, mostly commonly in the planning and delivery of prevention interventions. This is a promising sign of change to come.

Our key message is the importance of a whole-of-university approach. We have found that whole-of-university approaches are becoming more common, with social-ecological models guiding complex bundles of interventions that target the social, structural, and systemic determinants of campus-based sexual violence. Such an approach requires strong leadership and collaboration within, across, and beyond universities, informed by evidence of good practices and predicated on a commitment to rigorous evaluation.

To be comprehensive, the approach also necessitates multiple strategies for prevention and response that work *in concert*, bolstered by detailed monitoring and evaluation. Key examples of these strategies include appropriate, victim/survivor-led, person-centered, and trauma-informed pathways for support-seeking and reporting, as well as context-sensitive prevention interventions. Bringing these efforts together can better position universities to prevent violence before it occurs (primary prevention), work with campus community members at greater risk of experiencing or enacting violence (secondary prevention), and ensure continuity of care when the impacts of violence become chronic (tertiary prevention).

### Future Research, Practice, and Policymaking

Addressing campus-based sexual violence is long-term work. As a sector of higher education across Australia and Aotearoa New Zealand, we are just at the beginning. While we eagerly anticipate new evidence, we note here some crucial considerations for future research, practice, and policymaking. These considerations are informed by our reflections on what appears to have made differences across the four institutions included in our research. They also broadly correspond with the recommendations arising from *Change the Course*.

Two of our considerations are structural in nature and institution-wide in scope. First, we advocate for more humanized and flexible reporting pathways, with a stronger focus on promoting victim/survivors’ agency and choice. Second, we advocate for institution-wide, community-informed policy interventions that drive more ambitious agendas in addressing campus-based sexual violence. Examples include strategic frameworks, action plans, and other measures that drive institutional accountability. We argue that these structural changes are vital in supporting better outcomes for both individuals (such as through person-centered and trauma-informed mechanisms for sharing experiences and seeking action) and systems (such as through reorienting processes across the institution).

Our third consideration concerns resourcing. We advocate for secure support from senior leadership of higher education institutions. Scholars have discussed how precarious and inactive leadership can hamper efforts to address sexual violence in institutions of higher education (for example, see Bondestam & Lundqvist, 2020; Campbell et al., 2022; Linder & Myers, 2018). We posit that secure, supportive senior leadership can influence better outcomes in several ways, such as more appropriate funding and staffing for longitudinal efforts in prevention and response.

We fourthly advocate for universities to look beyond themselves in addressing the complex health and social issue of sexual violence. Partnerships with external organizations and services can enrich efforts—such as codesigned prevention that brings together universities’ resources and community organizations’ grassroots expertise. In some cases, external partnerships are necessary, such as referrals to longer-term support. These partnerships locate universities as part of wider communities—not just as settings for opportune prevention and response.

In tandem with the above, our fifth consideration recognizes that universities are unique contexts that demand tailored practices; one size does not fit all. We advocate for prevention and response efforts that are directly responsive to the specific strengths, needs, and ethos of each university. This is key when considering how universities can differ across our region—such as by regionality, size, student and staff demographics, rates of students’ commuting, institutional culture, and degree of involvement with surrounding communities.

Our final consideration focuses on evaluation. We advocate for more rigorous evaluation of practices, and for wider dissemination of findings, to help our sector learn. Evaluating multiple interventions can be difficult—especially if interventions are interdependent in achieving change. Evaluation must span the short, medium, and long term, with an analysis of how the undergirding theory of change has contributed to impacts and outcomes (Funnell & Rogers, 2011; Rogers, 2008). Given the heterogeneity of university communities, one beneficial approach would be realist evaluation (for example, see Westhorp, 2014)—exploring what worked, for whom, and under what conditions.

We also wish to contextualize our discussion within current world events. Universities can face unexpected change and disruption with deep impacts throughout campus communities. The COVID-19 pandemic is one such example. We are conscious of how the pandemic has affected university students’ lives; beyond sexual violence, scholars have also reported adverse impacts on students’ mental health (for example, see Cao et al., 2020; Sahu, 2020) and financial wellbeing (Montacute & Holt-White, 2020). Moreover, sexual and other forms of gender-based violence—such as domestic violence, family violence, and intimate partner violence—have not abated in wider society; evidence in fact suggests increased incidence (for example, see Boserup et al., 2020; Usher et al., 2020). We argue that universities must attend more greatly to these impacts as part of creating safer and more supportive environments.

## Conclusion

Universities are responsible for ensuring safety and wellbeing among students and staff. Despite this, we are concerned that institutional commitment to addressing campus-based sexual violence is at risk of weakening—particularly in the face of other health priorities. In a time of amplified public health action, we contend that universities must commensurately bolster their efforts in addressing campus-based sexual violence. This would help illustrate their arguably core commitments in community-building, health promotion and illness prevention, and social change. As such, buoyed by our findings and reflections, we believe this work to be more vital than ever.

We conclude with a call to action. Through our research, we aim to advocate for the continual work of addressing campus-based sexual violence throughout our region. We also seek to connect our experiences with the global community of researchers, practitioners, and policymakers who work to facilitate safe, respectful, and supportive university environments. Serendipitously, van Rensburg and Smith (2020) highlighted that the COVID-19 pandemic further compounded issues of outreach, access, and lack of evidence in the already precarious work of addressing campus-based sexual violence. Their reflection resonates with us as practitioners and researchers. Our collaboration—with this research being one of many outcomes—has allowed us the privilege of learning from each other. We call on further practitioners, researchers, teams, and institutions to join the conversation, as we continue to grow practice, shape policy, and build the evidence base—all crucial in responding to the shared crisis of sexual violence throughout universities across Australia and Aotearoa New Zealand.

## Appendix A

**Glossary of Region-Specific Terms**

**Table:**

**Term**	**Definition**
Australasian Universities Safer Communities Symposium	The Australasian Universities Safer Communities Symposium is a conference focused on practices that address campus community safety. Submissions are open to universities across the Asia-Pacific region.
Employee Assistance Program (EAP)	Employee Assistance Programs (EAPs) are designed to improve mental, emotional, and general psychological wellbeing among employees in organizations, including services for immediate family members. More information can be found at https://eapaa.org.au/site/.
Enhanced Assess, Acknowledge, Act (EAAA)	The EAAA program is designed to empower self-identified women to resist acquaintance sexual assault and to trust their judgment when their sexual safety is threatened. More information can be found at http://sarecentre.org/.
Epigeum	Epigeum, formerly part of Oxford University Press and now part of Sage Publications, has partnered with universities, and liaised with leading experts, across the United Kingdom and Australia to develop online courses relating to affirmative consent, responding to disclosures of sexual violence, and student health and wellbeing. More information can be found at https://www.epigeum.com/courses/support-wellbeing/.
Equity and Diversity	Refers to institutional departments, programs, and policymaking intended to support students and/or staff who have been underrepresented or in minority—including, but not limited to, people of different ages, races and ethnicities, abilities and disabilities, and genders and sexualities.
Full Stop Australia	Full Stop Australia is an Australian organization that supports people affected by sexual, domestic, or family violence. More information can be found at https://fullstop.org.au/.
Good Night Out	Good Night Out provides training on understanding and responding to sexual assault and sexual harassment in licensed premises. More information can be found at https://www.goodnightoutcampaign.org/info/.
NSW Police Area Command	NSW Police Area Commands form the local, community-based policing service in the Australian state of New South Wales. They are where most officers work as general duties police, detectives, highway patrol officers, and in traffic services. More information can be found at https://www.police.nsw.gov.au/about_us/regions_commands_districts.
NSW Sexual Violence Helpline	The NSW Sexual Violence Helpline, coordinated by Full Stop Australia, is for anyone in NSW who has experienced sexual assault; family members or other supporters of anyone who has experienced sexual assault; and others who have been impacted by this violence, including professionals. More information can be found at https://fullstop.org.au/get-help/our-services.
Respect. Prevent. Respond.	Respect. Prevent. Respond. is an Australian conference which encourages institutions to share ideas and initiatives within the context of preventing sexual violence on campus, building on what has been learned post-*Change the Course*.
*Sex, Safety & Respect*	The *Sex, Safety & Respect* program was developed by staff at Full Stop Australia. It facilitates education for the prevention of gender-based and sexual violence, focusing on consent, ethical communication and negotiation in interpersonal relationships, and bystander intervention. More information can be found at https://fullstop.org.au/training/for-students-and-educators/training-sex-safety-respect.
